# Digital Forensic Case Studies for In-Vehicle Infotainment Systems Using Android Auto and Apple CarPlay

**DOI:** 10.3390/s22197196

**Published:** 2022-09-22

**Authors:** Yeonghun Shin, Sungbum Kim, Wooyeon Jo, Taeshik Shon

**Affiliations:** 1 Department of Artificial Intelligence Convergence Network, Ajou University, Suwon 16499, Korea; 2 Department of Computer Science, Virginia Commonwealth University, Richmond, VA 23221, USA; 3 Department of Cybersecurity, Ajou University, Suwon 16499, Korea

**Keywords:** infotainment system, Android Auto, Apple CarPlay, vehicle forensics

## Abstract

Vehicle systems have been one of the fastest-growing fields in recent years. Vehicles are extremely helpful for understanding driver behaviors and have received significant attention from a forensic perspective. Extensive forensic research was previously conducted on on-board vehicle systems, such as an event data recorders, located in the electronic control unit or manufacturer-based infotainment systems. However, unlike previous vehicles that used only manufacturer-based infotainment systems, most vehicles today are equipped with infotainment systems such as Android Auto and Apple CarPlay. These in-vehicle infotainment (IVI) systems connect to mobile devices such as smartphones and tablets. The vehicle can periodically communicate with a smartphone and thus a network outside the vehicle. Drivers can use more services in their vehicles than ever before. Accordingly, an increasing number of diverse data are being stored in vehicles, with mobile devices connected to both the vehicle and the cloud. Such data include information that can be of significant help to investigators in solving problems during forensic investigations. Therefore, forensics of IVI systems such as Android Auto and Apple CarPlay are becoming increasingly important. We analyzed various forensic studies conducted on Android Auto and Apple CarPlay. Most of the research was mainly focused on mobile devices connected through a wired USB connection. The use of wireless-based IVI systems has recently been increasing. However, the analysis of Android Auto and Apple CarPlay from this point of view is insufficient. Therefore, we proposed a forensic methodology that fully considers such limitations. A forensic analysis was conducted on various IVI systems. We also developed an IVI system forensics tool that works based on the proposed methodology.

## 1. Introduction

With the development of various vehicle technologies, in-vehicle infotainment (IVI) systems are rapidly changing. Current IVI systems support connectivity with mobile devices, such as smartphones and tablets, and provide various services to drivers. The structure of such systems is also changing. Previously, vehicles were installed with only manufacturer-based forms of IVI systems, such as Ford Sync. However, in addition to manufacturer-based IVI systems, Google’s Android Auto and Apple CarPlay, first released in 2015 and 2014, respectively, are also being installed by default as of 2021. These systems work in connection with the driver’s mobile devices. When an Android or iOS device is connected to the vehicle, Android Auto and Apple CarPlay provide a function mirroring the mobile device environment to the vehicle. This allows the driver to use apps installed in the mobile device environment on the in-vehicle display. Such IVI systems are installed in vehicles developed by most manufacturers.

The vehicle is now connected to the driver’s mobile devices via the IVI system and communicates with an external network. This differs from the existing system, which only communicates various aspects within the vehicle [1]. IVIs such as Android Auto and CarPlay are connected to mobile devices and external networks, providing drivers with more information and services than ever before. However, the connection between the vehicle and mobile devices means that various information related to the driver or vehicle is transmitted between the two devices. Some of this information is stored on the IVI system or on a mobile device connected to the vehicle. 

Recently produced vehicles support a wireless connection between the IVI system and a mobile device, in order to provide convenience for the driver. In the case of a wireless communication environment there is a high risk of being exposed to various vulnerabilities. For example, if a vehicle’s wireless communication does not have a secure element such as authentication or encryption applied, an attacker can easily modify a message and it will be difficult for the devices to detect the modification [2]. Therefore, the importance of forensics in the wireless communication environment of vehicle infotainment systems is increasing. In addition, as IVI technology advances the scope of mobile devices forensics is expanding to include IVI, and like mobile devices, so vehicles are becoming an important source of digital evidence. 

Forensic research on vehicles has been conducted from two perspectives: a vehicle on-board system, and an IVI system. Forensic research on the on-board system of a vehicle has mainly been focused on an event data recorder (EDR) existing in the form of a module inside the electronic control unit (ECU) [3,4,5]. Most research on IVI systems have been forensic studies on manufacturer-based IVI systems [6,7,8,9,10,11]. Some forensic studies in recent years have also been conducted on the most popular IVI systems, i.e., Android Auto and Apple CarPlay [12,13,14,15]. However, these forensic studies are limited to the internal storage of mobile devices connected to Android Auto or Apple CarPlay systems, and the wireless connections they have recently begun supporting were not considered. 

As described above, Android Auto and Apple CarPlay systems work in conjunction with the driver’s mobile device. The connected mobile devices communicate with the vehicle using the protocol provided by the manufacturer of each IVI system. When communication outside the vehicle is required, the network of the mobile device is used [16,17]. The modern vehicle infotainment system environment has at least one wireless connection, so appropriate acquisition methods and analysis techniques must be applied for each wireless connection [18]. Therefore, the forensic analysis of Android Auto and Apple CarPlay systems can be divided into four areas: wireless communication between the cloud and mobile devices; wireless communication between the vehicle and mobile devices; internal storage of the mobile device; and internal storage in the IVI system. However, only a forensic analysis of the internal storage of the mobile device is currently conducted, and a forensic analysis of the remaining three areas remains insufficient. As a result, a forensic case investigation may be unable to obtain important information for resolving a particular case. We therefore propose a forensic methodology for a total of four areas including the internal storage area of a mobile devices, as previously performed. In addition, various IVI systems including real vehicles are used to validate the proposed forensic methodology. Based on the forensic methodology, we developed a digital evidence acquisition tool that helps with the forensics of IVI systems. The main contributions of this study are as follows:

1. We proposed a methodology for conducting IVI forensics. The proposed methodology consists of four analysis areas: wireless communication between the cloud and mobile device, wireless communication between the IVI and mobile device, internal storage of the mobile device, and internal storage of the IVI system. Excluding internal storage of a mobile device, the other three areas of analysis are newly proposed;
2. Eight IVI systems from various vehicle vendors are used as the case studies. As a result of a forensic analysis, various digital forensic artifacts were obtained in each analysis area. The artifacts we acquired can be used as reference materials in digital forensic investigations of IVI systems;
3. We developed a tool to acquire digital forensic artifacts from an IVI system, which operates based on our proposed forensic methodology. The tool was developed using a web-based application and automates the acquisition of digital forensic artifacts in four areas of forensics.

The rest of this paper is organized as follows: Section 2 reviews the existing research on vehicle systems; Section 3 describes the forensic methodology for IVI systems using Android Auto and Apple CarPlay; Section 4 presents case studies conducted on various IVI systems based on the proposed forensic methodology; Section 5 describes the limitations and implications of this study.; finally, Section 6 summarizes the study and discusses areas of future research.

## 2. Related Research

Forensic studies on vehicle data have been researched mainly from two perspectives: on-board and IVI systems. Forensic research of on-board systems has been conducted based on data acquired through the On-Board Diagnostic version II (OBD-II) protocol or data acquisition from storage devices such as hard disk drives (HDDs). Forensic research on IVI systems has been focused on data acquisition from a storage device such as the HDD of the IVI system, or mobile devices connected to the IVI system.

Previous studies of on-board systems have shown that vehicles store a variety of information, including the driver’s habits, locations, and driving time. Colin et al. noted the cybersecurity vulnerabilities of existing vehicle technologies and the importance of forensic research [5]. 

According to a study by Sladović et al., the RAM of an on-board system stores information such as the acceleration and speed during vehicle operation, and the last 5 s are stored in the EDR when a vehicle crash occurs. In addition, OBD-II and ECU/EDR connection are methods used to acquire the stored data. Moreover, Bosch’s Crash Data Retrieval or Berla’s iVe tool can be used for acquiring data stored in an on-board system [3].

Mekki et al. noted that data stored in a vehicle play an important role from a forensic perspective. Therefore, they proposed a neural network model for identifying drivers based on data stored in the vehicle. The data used to train the proposed model are also obtained through the OBD-II protocol [4]. 

Various studies have been conducted using on-board vehicle systems, the results of which can be fully utilized as a basis for vehicle forensics. However, such studies have not considered the use of an IVI system. 

An IVI system contains as much information as the on-board system. According to Henry, vehicle on-board systems can determine a point of impact, whereas IVI systems can show the longer-term driving habits of the vehicle’s driver. Henry used Berla’s iVe tool to analyze the Human–Machine Interface module of a 2015 Silverado pickup truck. Through this analysis, the author obtained various driver-related data, including a list of devices connected to the IVI system, phone calls, SMS messages, and certain GPS information [6]. 

In a study by Le-Khac et al., existing vehicle forensic studies were investigated, and case studies were conducted on multimedia devices of Volkswagen and BMW vehicles [10]. In a study by Lacroix et al., forensics of the first and second generations of Ford’s IVI system, SYNC, were described. Artifacts such as a list of connected devices and the phone book were acquired [7]. 

In a study by Cohen, forensics were also conducted on the third generation of SYNC. In this case, artifacts such as Bluetooth connections and driving and location information were acquired from NAND flash and an HDD [8]. 

In a study by Moos et al., the applicability of commercial forensic tools was analyzed for an HDD acquired from a BMW IVI system [9]. 

Ebbers et al. described a forensic study of a manufacturer-based legacy IVI app. They connected with the vehicle through a mobile app provided by the manufacturer and collected personal information stored on a smartphone. Experiments were conducted on a total of 10 manufacturers and their vehicles, and various information such as the vehicle identification numbers, driver data, and recent locations were obtained by analyzing Android and iOS devices [11]. 

Although various studies have been researched on IVI systems, Android Auto and Apple CarPlay systems, which have recently been installed in most newer vehicles, have not been considered.

With the popularization of Android Auto and Apple CarPlay among IVI systems, the importance of forensics in this area has grown, and some forensic studies have been presented. In Hickman’s Android Auto forensic study, data stored on a mobile device connected to Android Auto were acquired and analyzed. Using this method, the vehicle connection time, last location information, and Google Assistance records from Android Auto were acquired [13]. 

In a study by Edwards et al., a general mobile forensic technique was applied to mobile devices linked with Android Auto and Apple CarPlay, and artifacts such as the usage history and location information were acquired [12]. 

Following this study, Mahalik researched Android Auto connections through Bluetooth (BT) on an Android device. In addition, she analyzed various partitions in the mobile device to obtain records related to Android Auto and BT connections [15]. 

In another forensic study by Hickman, data stored on mobile devices connected to Apple CarPlay were acquired and analyzed, through which the vehicle linkage time and the last Siri usage record were acquired [14]. 

Various studies have been conducted on Android Auto and Apple CarPlay. Most such studies have applied mobile devices such as a smartphone and tablet. Modern IVI systems are connected to mobile devices, and thus forensics on mobile devices has gained importance. However, research in this area remains insufficient, and when conducting a forensic analysis of these IVI systems, consideration must be given to how they operate. The IVI system communicates with networks outside the vehicle by connecting to a mobile device. For wireless-based IVI systems, communication between each device is more exposed to the outside world. Communication between devices means that each device stores some of the communication data; however, not all communication data are usually stored on each device. Therefore, a forensic analysis of all communications in the IVI system must also be conducted. In addition, an analysis of the internal storage of IVI systems connected with Android Auto and Apple CarPlay remains insufficient.

## 3. Forensic Methodology for IVI Systems

**Figure 1:**
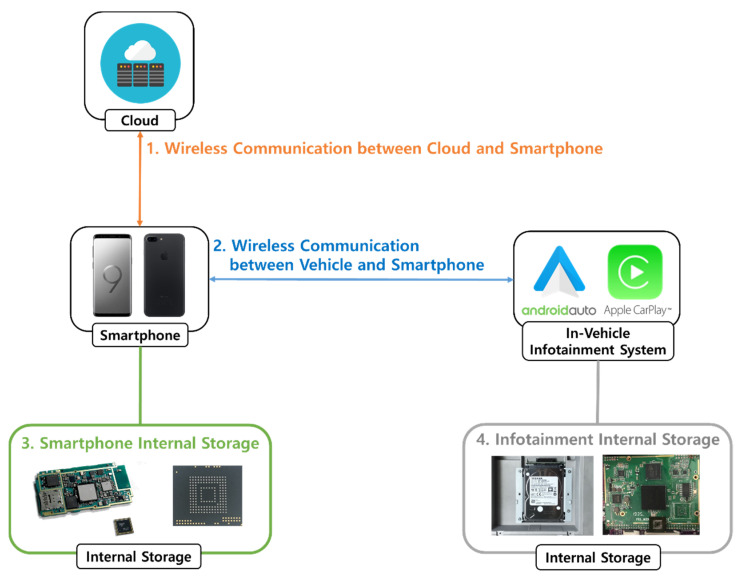
Forensic methodology for analysis of IVI system.

We propose a forensic methodology for IVI systems using Android Auto and Apple CarPlay. Figure 1 represents the forensic methodology and IVI environment. The proposed forensic methodology reflects the characteristics of the current IVI environment, which consists of the in-vehicle IVI system, the driver’s mobile device, and the cloud for providing IVI services. To use the IVI system, the driver must connect between the IVI system and the mobile device. There are two types of connection between these two devices: wired and wireless. A wired connection uses USB, and a wireless connection uses Wi-Fi and BT. For the communication method, different protocols are used depending on the manufacturer of the IVI. For Apple CarPlay, some communication methods are open to the public [19]. When the IVI system and mobile devices are connected, periodic communication between the two devices is conducted. This communication includes information related to the vehicle or driver, and some of this information is stored in the internal storage of the driver’s mobile device or the IVI system. In addition, the IVI environment communicates with the cloud server outside the vehicle to provide application services such as navigation. This communication takes place on the mobile device. To the best of our knowledge, a vehicle cannot currently communicate with the cloud on its own. 

The above communication also includes personal information. For example, if the driver is using a navigation app, the communication is likely to include the current location or destination of the vehicle. Considering the characteristics of the IVI environment described above, we divided the forensic methodology into four areas of an IVI system are as follows:

1. Wireless communication between the cloud and mobile device;
2. Wireless communication between the IVI and mobile device;
3. Internal storage in the mobile device;
4. Internal storage in the IVI system.

### 3.1. Wireless Communication between the Cloud and Mobile Device

IVI systems such as Android Auto or Apple CarPlay are connected to the mobile device, and the network availability of the mobile device is essential to remain online. When the vehicle and mobile device are connected, the IVI environment uses the network of the mobile device to communicate with a cloud server outside the vehicle. This communication, i.e., wireless communication between the cloud and mobile device, contains data directly related to the services used by the driver. Although unrelated to the communication between the vehicle and the mobile device, it has been proven that various artifacts can be obtained by analyzing the communication between the device and cloud [20]. 

Most of this communication is encrypted through the transport layer security (TLS). To analyze this, it is essential to apply a man-in-the-middle (MitM) by installing a certificate on the mobile device [21]. To analyze this communication and acquire important information, a certificate is installed on the mobile device in advance and proxy settings are configured. Web proxy tools such as Charles or Fiddler are used to acquire and analyze information on the communications.

### 3.2. Wireless Communication between the IVI and Mobile Device

IVI systems such as Android Auto or Apple CarPlay are connected to a mobile device to periodically communicate with each other. As mentioned earlier, this communication includes information related to the vehicle or driver. From previous studies of mobile devices connected with Android Auto or Apple CarPlay, it is known that various information regarding the use of these IVI systems is stored in the mobile device. However, there is still insufficient research on acquiring and analyzing such information in the communication between two devices. An analysis of this aspect is also important from a forensic perspective because it can help in gathering a variety of information. 

Wired and wireless methods are used for connecting Android Auto and Apple CarPlay systems to a mobile device, and this area of analysis focuses on the latter. In our experiments, Android Auto and Apple CarPlay use both BT and Wi-Fi for their wireless connections. Therefore, we collected and analyzed BT and Wi-Fi packets from Android and iOS devices. In addition, the packet analysis results using Android Auto and Apple CarPlay are compared with the analysis results of the connected legacy system without the application of the above IVI system. For acquisition and analysis on an Android device, Android’s own featured BT Snoop and Wireshark tools are used. For iOS devices, Xcode’s PacketLogger and Wireshark tools are applied.

### 3.3. Internal Storage in the Mobile Device

Previous studies have demonstrated that mobile devices connected to IVI systems such as Android Auto and Apple CarPlay store various information related to the driver and vehicle. Therefore, we analyzed the newly added artifacts in slightly more detail using the newer versions of the IVI system applications installed on a mobile device. 

In general, administrator privilege is basically required to obtain data stored in the internal storage of the mobile device [22]. Therefore, rooting of the Android devices and jailbreaking of the iOS devices were required. The internal storage was then dumped in the form of raw images, and general mobile forensic analysis techniques were applied. There is a possibility that some data will be deleted over time. Therefore, file recovery techniques must also be applied for each filesystem. Tools such as HxD, DB Browser for SQLite, FTK Imager, and the Sleuth Kit (TSK) are used for data collection and analysis.

### 3.4. Internal Storage in the IVI System

Previous studies have demonstrated that the IVI system stores information related to a connected mobile device, the driver, or the vehicle. However, there remains a lack of forensic studies on the internal storage of IVI systems using Android Auto and Apple CarPlay. In this analysis area, the internal storage of the IVI system using Android Auto and Apple CarPlay is acquired and analyzed. 

In general, the internal storage of IVI exists in the form of HDD or flash memory on a printed circuit board (PCB). If the internal storage of the IVI system uses an HDD, the data can be acquired by simply removing the drive from the vehicle. However, if the data are stored in flash memory, they must be accessed through an interface such as a Universal Asynchronous Receiver-Transmitter (UART) or Joint Test Action Group (JTAG). A chip-off may be applied as a final option if access is impossible owing to the security features of the IVI system manufacturer. As tools for data collection and analysis, HxD, DB Browser for SQLite, FTK Imager, and TSK are used for internal storage in the mobile device.

## 4. IVI Systems Forensic Use Cases

This section describes case studies conducted on actual IVI systems based on the proposed forensic methodology. The IVI system used in the case study can be divided into vehicle systems and navigation systems. We selected 8 IVI systems considering this classification, whether they support Android Auto and Apple CarPlay, and whether they support wireless connectivity. Two of the eight IVI systems are drivable vehicles and head units acquired from vehicles; the other six are navigation systems that can be purchased online. Table 1 shows the IVI systems used in case studies and the connection possibilities on Android Auto or Apple CarPlay. Connectivity is expressed as Not Supported (not-supported connection), Wired (supported wired connection), and Wireless (supported wireless connection). For IVI systems marked as Wireless, a wired connection is also supported. 

**Figure 2:**
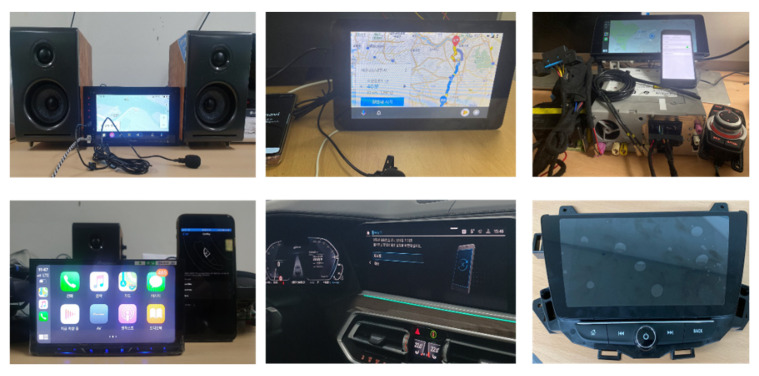
Test IVI environment for forensic analysis.

Table 2 shows the mobile devices and tools used in the experiment. Smartphones were used as the mobile devices connected to the vehicle. A Samsung (Suwon, South Korea) Galaxy S9 with Android 10 and an Apple (Cupertino, CA, USA) iPhone 7 with iOS 13.3.1 were used. Figure 2 shows six of the eight test IVI environments configured by connecting an IVI system and a mobile device (from top left: TTEC (Englewood, CO, USA) D5, Raspberry Pi 3B, BMW (Munich, Germany) NBT HU EVO, Pioneer (Bunkyo City, Tokyo, Japan) AVH-Z5050BT, BMW X5 45e xLine, and Chevrolet (Detroit, MI, USA) TrailBlazer). 

We tried to generate the same experiment data from the case studies of all IVI systems. After connecting the IVI system and the mobile device, we used the contact synchronization, phone calls, SMS messages, and use of the navigation application.

### 4.1. Wireless Communication between the Cloud and Mobile Device

The way a mobile device communicates with the cloud server differs depending on the connection method applied between the IVI system and the mobile device. When Android Auto and a mobile device have a wired connection, the mobile device communicates with the cloud server using Wi-Fi or Long-Term Evolution (LTE), and when connected wirelessly with the mobile device, the mobile device communicates with the cloud server using LTE. Regardless of whether Apple CarPlay and the mobile device are wired or wirelessly connected, the mobile device uses LTE to communicate with the cloud server. However, the MitM-based web proxy tool operates based on the Wi-Fi communication and does not support MitM for LTE communication. Therefore, this experiment can only be conducted using Android Auto. We collected and analyzed the Wi-Fi traffic of an Android device communicating with cloud servers. To apply MitM to Android smartphones, we referred to various previous studies [29,30,31,32]. A Raspberry Pi 3B with Craftshaft and TTEC’s D5 IVI systems were used for this experiment. 

**Figure 3:**
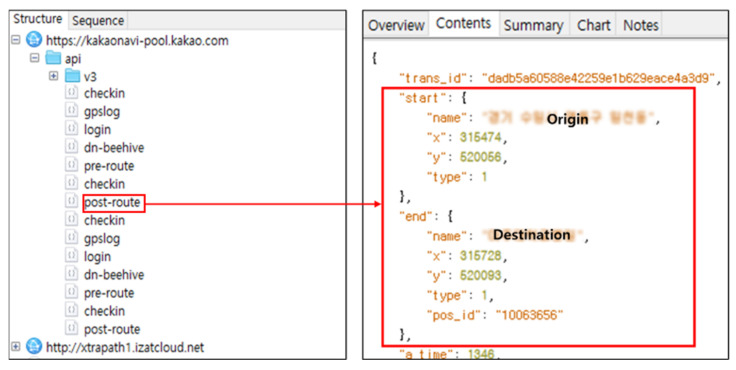
Communication traffic of navigation application.

We collected Wi-Fi traffic before the IVI system and the mobile devices were connected, after they were connected, and until the test data were generated. As a result of the analysis, no traffic types related to contact synchronization, SMS messages, or phone calls were identified. However, when the direction function of the navigation application was executed, we obtained traffic communication with the cloud server of the navigation application, as shown in Figure 3. This includes content that can determine the origin and destination of the driver. Some navigation application manufacturers may have stored this information [33,34,35].

As a result of the analysis of wireless communication between the cloud and mobile device, four artifacts are acquired from the traffic of the navigation app: origin name, destination name, origin coordinate, and destination coordinates. Detailed artifacts are listed in Table 3. However, the artifacts we acquired are related to the third-party cloud, and not directly related to Android Auto.

### 4.2. Wireless Communication between the IVI and Mobile Device

In this experiment, we conducted a collection and analysis of communication packets sent and received by Android Auto or Apple CarPlay with a mobile device. To collect communication packets between the IVI system and a mobile device, an experiment was conducted in a wirelessly connected environment. When Android Auto and Apple CarPlay connect wirelessly with a mobile device, they communicate using both BT and Wi-Fi channels. Therefore, we collected traffic from BT and Wi-Fi channels. 

A BMW NBT HU EVO, a Pioneer AVH-Z5050BT, and a BMW X5 45e xLine were used in this experiment. BMW NBT HU EVO only supports Apple CarPlay wireless connection and does not support wireless connections to Android Auto. The Pioneer AVH-Z5050BT does not originally support a wireless connection to Apple CarPlay; however, a wireless connection is available using a CPC200-U2W PLUS adapter from CarLinkit Factory(. The BMW X5 45e xLine is the only one of the IVI systems we tested that supports wireless connectivity for both Android Auto and Apple CarPlay. To collect BT traffic of Android Auto, we used BT Snoop, a feature provided by Android OS. The Xcode PacketLogger tool was used to collect BT traffic from Apple CarPlay and a Wireshark tool was used to collect Wi-Fi traffic from both systems.

In addition, we applied BT connections between mobile devices and legacy systems that do not use Android Auto or Apple CarPlay and collected traffic from the BT channels. Both Android Auto and Apple CarPlay use BT and Wi-Fi for wireless connections. However, the connection of a legacy system also uses a BT channel. It is important to compare the traffic generated by the BT channel of Android Auto or Apple CarPlay with the traffic generated by the BT channel of a legacy system. Based on this comparison, we can determine whether the actual usage data of Android Auto and Apple CarPlay are also transmitted over the BT channel, or only through the Wi-Fi channel. Unlike Android Auto and Apple CarPlay, the legacy system does not mirror the navigation application of the mobile device, and thus only the functions of contact synchronization, SMS messages, and phone calls were used.

As a result of analyzing the BT traffic of Apple CarPlay, various artifacts and communication specifications were acquired. Artifacts that could be obtained on all devices were almost the same. It was confirmed that the BT communication of Apple CarPlay mainly uses an Audio/Video Remote Control Profile, host controller interface, and radio frequency communication (RFCOMM) protocols. It was also confirmed that some AT commands are used. As the result of a detailed analysis, details such as the BT address, BT name, pairing pin, encryption key, and related information of the vehicle and mobile device were acquired. In addition, the International Mobile Equipment Identity (IMEI) and International Mobile Subscriber Identity (IMSI) of the mobile device were acquired. Artifacts could also be acquired from Wi-Fi communication; however, the analysis was difficult in most cases because the traffic was encrypted.

**Figure 4:**
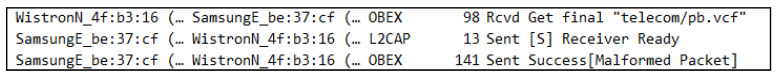
Contact synchronization packets in Android Auto.

As a result of analyzing the BT traffic of Android Auto, we obtained artifacts and communication specifications similar to those of Apple CarPlay. Android Auto also uses communication protocols over L2CAP channels such as RFCOMM and audio/video distribution transport protocol (AVDTP). Unlike Apple CarPlay, it uniquely uses the object exchange (OBEX) protocol for synchronizing the contacts. Figure 4 shows the process of requesting and receiving the contact file pb.vcf (phonebook.vcf) through the OBEX protocol. Using this packet, the contact stored in the mobile device can be obtained. In addition, artifacts such as the BT address and BT name were acquired. Unlike Apple CarPlay, the IMEI and IMSI of the mobile device cannot be obtained. In the case of Wi-Fi traffic, data are transmitted through [PSH, ACK] when the buffer is full. However, it appears to use encryption, and thus the content cannot be analyzed from every packet. Detailed artifacts are listed in Table 4.

**Figure 5:**
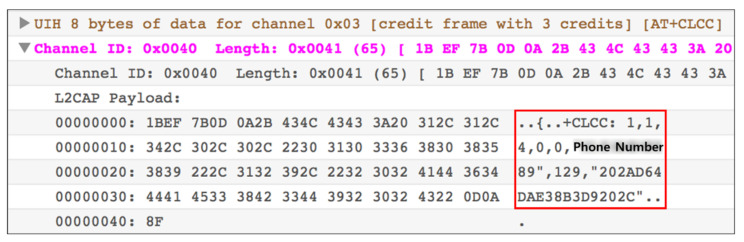
Phone number acquired in BT traffic (legacy system). From the red frame, the phone number and name saved in contacts are obtained.

As a result of conducting an analysis on the legacy system, various artifacts and communication specifications were obtained. The same protocol is used for BT communication with both Android Auto and Apple CarPlay. However, more AT commands are used in the legacy system. As a result of the detailed analysis, in addition to the existing artifacts, details of the phone number of the call recipient and the phone number of the caller were also acquired. Figure 5 shows the acquired phone number. Comparing the artifacts acquired in the legacy system with the system using Android Auto and Apple CarPlay, it is assumed that they are transmitted over the Wi-Fi channel, excluding the initial pairing and contact synchronization. Detailed artifacts are listed in Table 5.

### 4.3. Internal Storage in the Mobile Device

Forensic research on mobile devices connected to Android Auto or Apple CarPlay has been actively conducted. According to known studies, the mobile device stores the connected vehicle information, IVI system usage history, and location information [12,13,14,15]. Therefore, when conducting the forensic analysis of mobile devices connected to a vehicle, we focused on finding newly added artifacts in the latest version of the IVI system. Mobile data acquisition and forensic analysis methods and forensic procedures were performed based on methods proposed in various studies conducted on Android OS and iOS [36,37,38,39,40,41]. Our analysis results can be used as a basis for future forensic investigations targeting Android Auto or Apple CarPlay systems. 

BMW NBT HU EVO, BMW X5 45e xLine, Sony XAV-AX5000, and Pioneer AVH-Z5050BT were used in the experiments. When collecting data stored in the internal storage of a mobile device, the data were divided into cases of wired and wireless connections to the IVI system. For the Android Auto app, we used the latest version of Android 10, 7.1.614573 (as of December 2021, Mountain View, California, United States), on a Galaxy S9. For the Apple CarPlay app, the latest version of iOS, 13.3.1 (as of December 2021, Cupertino, California, United States), was used on an iPhone 7.

Analysis of data acquired from the internal storage of a mobile device connected with Android Auto confirmed that most of the artifacts acquired in the previous study were obtainable. However, we also discovered new artifacts added in the latest version of Android Auto. The newly added files are carservicedata.db and carservice.xml, which are placed inside the app directory of Android Auto. 

**Figure 6:**
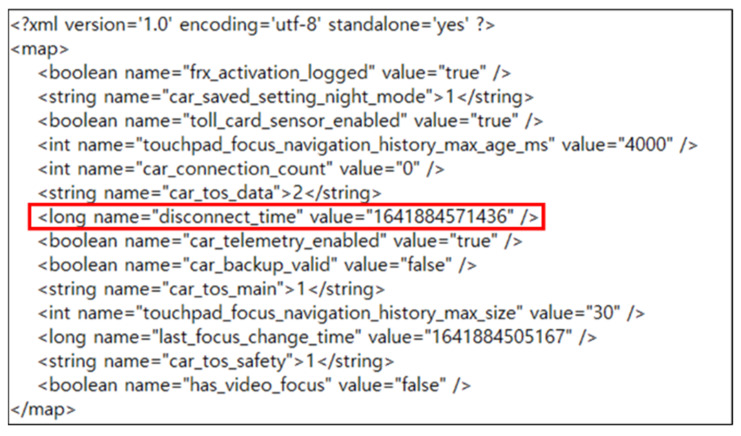
The disconnect_time field inside the carservice.xml file. The red frame indicates the disconnection time (disconnect_time).

Among them, carservicedata.db is a file that originally existed in the com.google.android.gms directory, according to a previous study [15]. This includes information such as the vehicle model, vehicle ID, and vehicle connection time. Because it stores information on connected vehicles, it appears to have been recently moved to the Android Auto package. The carservice.xml is a newly added file that stores various types of information. From a forensic perspective, however, the important information is the vehicle disconnection time (disconnect_time). Figure 6 shows the carservice.xml. 

In addition, there is a file that already exists in the Android Auto package, although its contents have been changed. A new mirroring start time (pref_projected_activation_date) field has been added to the app_state_shared_preferences.xml file. An analysis of the data acquired from the internal storage of mobile devices connected to Apple CarPlay confirmed the results of previous studies. Detailed artifacts are listed in Table 6.

### 4.4. Internal Storage in the IVI System

In order to analyze the internal storage of the IVI system to obtain meaningful results from a forensic point of view, sufficient data must be stored in the target IVI system. Therefore, this experiment was conducted on the IVI system in which sufficient data was stored by applying the previous three case studies. 

We conducted an analysis on six out of the eight IVI systems. Among the IVI systems we tested, a BMW X5 45e xLine was used for testing the wireless connectivity on Android Auto. Therefore, this IVI system was excluded from the experiment. We also excluded the crankshaft installed on the Raspberry Pi 3B device. Each IVI system was disassembled to check for the existence of an HDD, and if one was present, it was separated from the IVI system. We also analyzed the PCB to find a way to acquire data from flash memory. We minimized the damage when chip-off the flash memory from the PCB by referring to previous studies [42,43].

**Figure 7:**
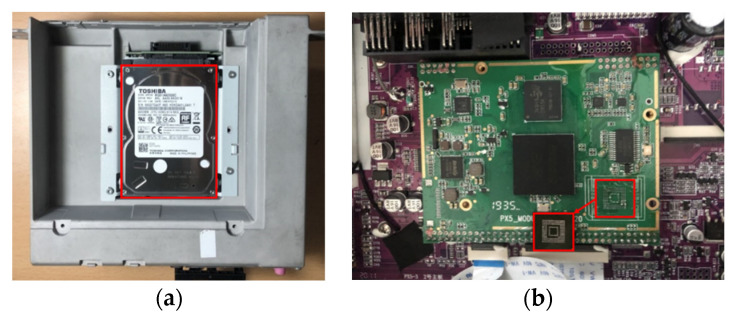
The red frames indicate the storage of the IVI system: (**a**) HDD acquired from BMW NBT HU EVO, (**b**) NAND flash acquired from Belsee Best Aftermarket Auto.

As a result, internal storage was obtained from a BMW NBT HU EVO and Belsee Best Aftermarket Auto. In the case of the BMW NBT HU EVO, an HDD is used. Belsee Best Aftermarket Auto has a flash memory capable of a chip-off. For other IVI systems, an analysis was conducted by considering UART and JTAG access; however, there were difficulties owing to the security features of the manufacturer. Figure 7 shows the HDD acquired from the BMW NBT HU EVO and the flash memory acquired from the Belsee Best Aftermarket Auto.

We analyzed the acquired BMW HDD and the Belsee flash memory. Table 7 lists the specifications for both types of internal storage. To acquire the data from the HDD of the BMW, we mounted the drive to a PC. However, it was confirmed that the HDD of the BMW system was ATA locked. To solve this problem, we refer to various studies related to BMW HDD and ATA and articles for unlocking the ATA Lock of BMW HDD [9,44,45]. However, in the case of the BMW NBT HU EVO system used in the experiment, with a higher level of security than before, analysis could no longer be performed.

**Figure 8:**
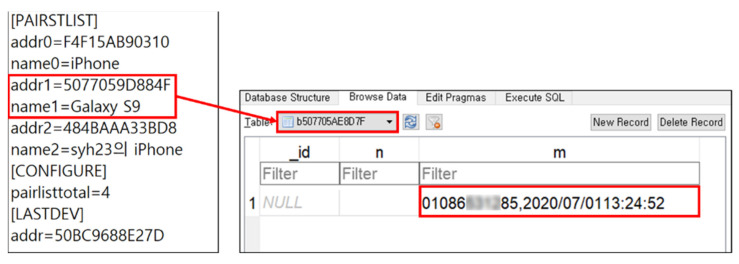
BT paired device list (bt_conf.ini) and phone number acquired from Belsee Best Aftermarket Auto (DateBase1.db).

We conducted an Android-based forensic analysis on the internal storage acquired from the Belsee IVI system. A forensic analysis of the Ext filesystem was conducted based on our existing studies [46,47], and a forensic analysis of the F2FS filesystem was conducted using a TSK-based tool developed by our team. As the analysis results indicate, the vehicle connection and usage information were acquired from the internal storage. In addition, we confirmed that the drive contained artifacts that are commonly acquired on an Android device. Among them, only artifacts related to the Belsee IVI system and directly related to the mobile device were considered. Figure 8 provides a list of mobile devices connected to the Belsee IVI system. The left side of Figure 8 shows the file recording of the BT address and the BT name of the connected mobile devices (bt_conf.ini); the right side shows the database file with the BT address values in a table form (DateBase1.db). In this database file, the phone number and connection time of the mobile device connected to the vehicle are recorded. By correlating these two files, you can obtain BT-related information and phone number of the connected mobile device. In addition, we acquired artifacts on the locations where the vehicle was connected. Detailed artifacts are listed in Table 8.

### 4.5. Cross-Validation of In-Vehicle Infotainment System Artifact

We conducted experiments on four methods for collecting forensic artifacts from the In-Vehicle Infotainment experimental environment and real vehicles. Based on this, we collected various forensic artifacts. Collected artifacts can be correlated to assist in forensic investigations. 

In actual forensic investigations, Section 4.1 and Section 4.2 tend to be real-time, so they are not generally suitable for post-forensic investigations. If it is not a planned investigation of a specific person, this corresponds to the prior knowledge that investigators should know before conducting a forensic investigation into the In-Vehicle Infotainment System. Therefore, in the post-forensic investigation, cross-validation can be performed for Section 4.3 (Internal Storage in the Mobile Device) and Section 4.4 (Internal Storage in the IVI System).

Before discussing methods for performing cross-validation on collected forensic artifacts, these are just a few of the many examples that can be used for forensic investigations. The forensic artifacts collected using the method proposed in this paper can be utilized in a variety of other ways besides the few examples we mention. We have summarized three methods for performing cross-validation on forensic artifacts collected based on the proposed method:

1. Forensic investigators can examine the driver’s smartphone to determine which apps the driver used in Android Auto or Apple CarPlay. Based on this, when performing analysis on infotainment internal storage, it is possible to reduce the time required for forensic investigation by determining the main analysis target in advance;
2. Forensic investigators can examine the driver’s smartphone to determine vehicle information related to usage time. Information that can be identified includes when the driver last used Android Auto or Apple CarPlay, and the connected vehicle’s Bluetooth Name and MAC Address. Afterwards, forensic investigators can perform an analysis on infotainment internal storage to obtain a Bluetooth Log. Based on this, the forensic investigator can determine the vehicle’s Bluetooth Name and MAC Address, as well as information about the smartphone connected to the vehicle. It is possible to specify a suspect by combining artifacts collected from these two sources. This can also be done in reverse order;
3. If a planned investigation is carried out on a specific person, sniffing for wireless communication between vehicle and smartphone such as Bluetooth and LTE may be performed. In this case, the Bluetooth Name and MAC address of the vehicle and smartphone can be identified through the wireless communication section packet. In addition, it is possible to obtain the driver’s call record and a unique identification number such as the IMEI of the smartphone. This information can help specify the driver’s smartphone in a post-forensic investigation and provide a non-repudiation. Also, when a forensic investigator performs an analysis on the Smartphone internal storage, only the last connected time can be obtained. However, based on the packet information collected in advance, the forensic investigator can timeline the time information when the smartphone is connected to the Infotainment System.

### 4.6. Significant Artifacts from IVI System Analysis

We performed case studies for the actual vehicle environment. Most of the forensic studies on IVI systems that have been conducted are indirect forensic artifact collection studies limited to mobile devices. Accordingly, we conducted a forensic analysis of the internal storage of smartphones along with three new forensic analysis sections. As a result, we were able to acquire a variety of artifacts, including those acquired by existing studies. The acquired artifacts are summarized in Table 3, Table 4, Table 5, Table 6 and Table 8.

As a result of the analysis of Wireless communication between the cloud and mobile device, four artifacts related to the origin and destination were derived from the traffic of the navigation app. The derived artifacts are summarized in Table 3. In addition, it was confirmed that the artifacts derived from the Android Auto environment and Crankshaft are the same. 

As a result of our analysis of Wireless communication between the IVI and mobile devices, we obtained over 10 artifacts related to vehicles and smartphones. The acquired artifacts are summarized in Table 4 and Table 5. The acquired artifacts include vehicle information as well as information that is directly related to the connected smartphone. The derived artifacts have great significance in themselves, but they can also be used for cross-validation with other analysis sections. This can increase the reliability of the overall analysis results.

As a result of the analysis of Internal storage in the mobile device, five artifacts were derived from Samsung Galaxy S9+ devices, and four artifacts were derived from Apple iPhone 7 devices. We derived only artifacts that are directly related to the use of IVI systems, excluding information stored due to the use of a typical smartphone. The artifacts are summarized in Table 6. One of the artifacts acquired from Android Auto was a new artifact. This artifact serves as a reference for selecting an app to perform forensic analysis. In addition, we have shown that the acquired artifacts can be used to conduct organic analysis to determine the relevance of messaging and call records to vehicle use. Furthermore, based on our research, we confirmed that some artifacts that can be obtained may differ depending on the OS version. Android Auto does not exhibit much difference, whereas for Apple CarPlay, fewer artifacts can be acquired on the latest OS. In addition, the derived artifacts can be used for cross-validation with other analysis results. 

Analysis of the Internal storage in the IVI system revealed four artifacts from Belsee Best Aftermarket Auto. These are summarized in Table 8. Because it included the BT Name and BT Address of the smartphone connected to the vehicle, it was possible to perform cross-validation with artifacts derived from the analysis of the wireless communication between the vehicle and smartphone and the internal storage of the smartphone. In addition, we derived the vehicle usage time and location information artifacts that are difficult to obtain from a smartphone. As a result of the analysis of the BMW NBT HU EVO, the vehicle internal data could not be acquired due to the reinforced ATA lock set on the HDD. We were only able to derive the type of OS and filesystem used by the BMW NBT HU EVO.

### 4.7. Forensic Analysis Tool for IVI System

We developed a forensic tool that operates based on the four areas of the proposed forensic methodology. Therefore, our forensic tools provide a total of four functions: 

First, the analysis of traffic between mobile devices and the cloud is automated. Packets from mobile devices and the cloud are ingested through the Charles Proxy tool. The tool analyzes the collected packets to obtain information such as the origin and destination of the navigation app. 

Second, it automates the analysis of BT packets between the mobile device and the vehicle. When a BT packet between a mobile device and a vehicle is input into the tool, the tool parses the packet file to obtain the artifacts derived through the analysis. 

Third, it automates the analysis of the internal storage of the mobile device. Raw images obtained from the internal storage in the mobile device are imported into the tool, and the tool acquires the derived artifacts. In addition, it can organize the acquired artifacts based on the timeline. 

Fourth, it automates the analysis of the internal storage of the IVI system. In this case, because the analysis was conducted only on Belsee Best Aftermarket Auto devices, it only works on an Android-based IVI. When the tool applies a raw image from the internal storage of the IVI, the tool acquires the derived artifacts. In addition, it can also organize the acquired artifacts based on the timeline. 

**Figure 9:**
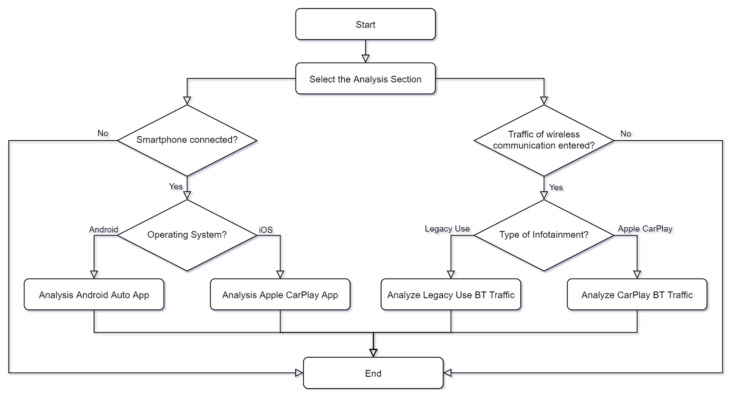
Flowchart for analyzing BT packets between a mobile device and a vehicle.

Figure 9 shows a flowchart of analyzing BT packets between a mobile device and a vehicle. The artifact acquisition and analysis automation tool works with the following procedures:

1. Select analysis target (Internal Storage of Smartphone(2) or BT packet(3));
2. Analysis of Smartphone’s Internal Storage
  2.1 After connecting the smartphone, select the operating system to be analyzed
  2.2 The analytics tool performs the process of collecting data from a specific location on the smartphone’s internal storage
  2.3 Analyze the collected data to derive forensic artifacts and output them as results;
3. Analysis of BT packet
  3.1 After entering the packet file, select the IVI system to be analyzed
  3.2 Parsing and analyzing the contents of the packet file to derive forensic artifacts and output them as results.

**Figure 10:**
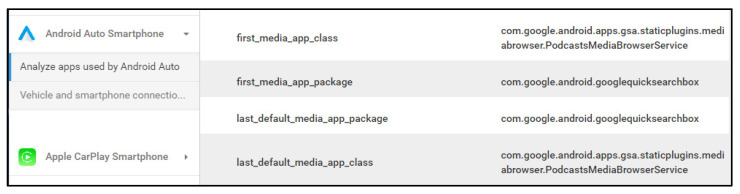
Results of analyzing the Internal Storage of Android Smartphone.

**Figure 11:**
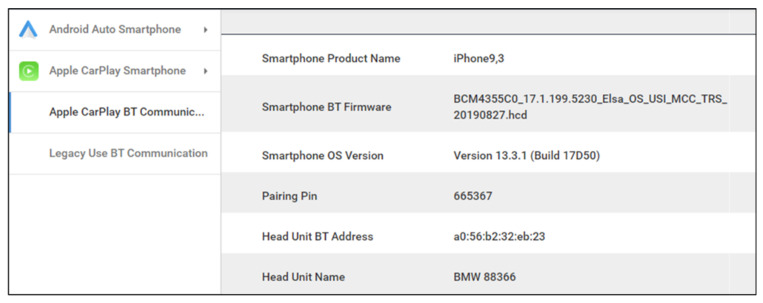
Results of analyzing the BT packet between the mobile device and vehicle.

Figure 10 shows the results of the tool developed for analyzing the Internal Storage of an android smartphone. The figure shows the default app setting of Android Auto and the last used app output as a result of analyzing the internal storage of the Android smartphone. Figure 11 shows the results of the tool developed for analyzing the BT packets between the mobile device and the vehicle. The figure shows that the developed tool analyzes the BT packet and outputs information about the connected smartphone, pairing pin, and information on the IVI system.

## 5. Discussion

Although forensic studies of the Android Auto and Apple CarPlay IVI systems have been conducted, the operation method of the IVI system was not taken into account in the forensic analysis. For this reason, there are limitations in the field of IVI system forensics that have made it difficult to collect forensic artifacts in some areas of analysis. To overcome these limitations, we proposed a forensic methodology that considers the operation of an IVI system and analyzed several different systems. Our findings show that a forensic analysis of wireless communication in an IVI system is meaningful. In addition, we found important information that is meaningful from a forensic perspective in the storage of a mobile device and the storage of an IVI system. We have overcome the limitations of IVI system forensics to a certain extent by applying the proposed forensic technique.

There are certain limitations to our case studies. The analysis of the wireless communication between the cloud and mobile device could only be conducted using Android Auto, because Apple CarPlay applies LTE for communication between the cloud and mobile devices. In the analysis of the wireless communication between the IVI and a mobile device, we compared the analysis results of the legacy system using Android Auto and Apple CarPlay. The results show that most of the actual usage data for Android Auto or Apple CarPlay were transmitted over the Wi-Fi channel. However, we could not analyze the Wi-Fi channel owing to encryption or the communication standards of the manufacturer. Nevertheless, forensic analysis results show the need for a wireless communication analysis in IVI systems. A detailed analysis of the internal storage in the IVI system could not proceed owing to encryption of the HDD of the BMW NBT HU EVO. 

Despite the above limitations in our case studies, we obtained sufficiently diverse forensic artifacts in the IVI environment. In particular, newly added artifacts were discovered in the analysis of the internal storage of the mobile device connected with Android Auto. These artifacts can be used as important evidence in conducting a forensic investigation because they contain timestamps that are connected to or disconnected from the vehicle. In addition, we analyzed the internal storage used in the IVI system based on the Android platform. Because the share of Android Automotive OS has been gradually increasing in recent years, the analysis results are also sufficiently meaningful.

## 6. Conclusions

Forensic studies of Android Auto and Apple CarPlay systems have only been conducted on the internal storage of mobile devices. However, there are also IVI systems that make up the IVI system environment. Therefore, analysis must also be performed on the data stored inside the IVI system, and since the IVI system and the mobile device are connected, the resulting communication section is also subject to analysis. Therefore, we have proposed a forensic methodology and conducted various case studies to address the shortcomings in the field of IVI system forensics research. The analysis results showed that the proposed methodology can solve the above constraint. In addition, the forensic artifacts acquired in each analysis area show that the forensic artifacts obtainable in IVI systems using Android Auto and Apple CarPlay are not limited to the internal storage in a mobile device. As such, a digital forensic examination of various elements constituting the IVI system is a useful addition to vehicle forensics. An increasing number of people are using Android Auto and Apple CarPlay, and more vehicles and manufacturers are sup-porting these systems. The proposed forensic methodology and the acquired artifacts can be applied as an important basis for future vehicle crime investigations.

As IVI technology advances, mobile devices and IVI systems are increasingly making use of driver-related information. In the near future, IVI systems will become an important source of evidence in digital forensic investigations, just like today’s smartphones. In our study, although the analysis of various sections constituting the IVI system environment was performed, there are still shortcomings. By performing decompilation analysis on IVI applications with future work, communication structure analysis studies on Wi-Fi communication channels of IVI systems can be performed. This allows you to perform a complete analysis of the communication between the IVI system and your mobile device. In addition, most of the IVI systems used for analysis in our study were chip-off resistant systems. Therefore, it is possible to conduct research on data acquisition methods using hardware debugging interfaces such as UART and JTAG for IVI systems that cannot be chip-off with future work.

## Figures and Tables

**Table 1:** IVI system used in case studies and connectivity of the IVI system.

Manufacturer	IVI System	Android Auto	Apple CarPlay
BMW	NBT HU EVO	No Support	Wireless
BMW	X5 45e xLine	Wireless	Wireless
Chevrolet	TrailBlazer	Wireless	Wireless
Pioneer	AVH-Z5050BT	Wired	Wireless
Sony	XAV-AX5000	Wired	Wired
TTEC	D5	Wired	No Support
RASPBERRY-PI	Raspberry Pi 3B (Crankshaft)	Wired	No Support
Belsee	Best Aftermarket Auto	Wired	Wired

**Table 2:** Specification of mobile devices and software used in case studies.

Manufacturer	Mobile Devices and Software	Version
Samsung	Galaxy S9+	Android 10
Apple	iPhone 7	iOS 13.3.1
Google	Android Auto [16]	7.1.614573
Apple	Apple CarPlay [17]	Depends on iOS version (13.3.1)
Crankshaft	Crankshaft [23]	csng-alpha 5.1
The Wireshark team	Wireshark [24]	3.4.0
Apple	PacketLogger [25]	7.0.0
Sublime HQ Pty Ltd.	Sublime Text 3 [26]	3.2.2
sqlitebrowser	DB Browser for SQ Lite [27]	3.10.1
Karl von Randow	Charles Web Debugging Proxy [28]	4.5.6

**Table 3:** List of Artifacts in Wireless Communication between the Cloud and Mobile Device.

IVI System	Artifact	Details
Android Auto	Origin	Woncheon-dong
	Destination	Ajou University Hospital
	Origin X, Y	315,474, 520,056
	Destination X, Y	315,728, 520,093

**Table 4:** List of Artifacts in Wireless Communication between the IVI and Mobile Device (Android Auto and Apple CarPlay).

IVI System	Artifact	Details
Android Auto	Vehicle BT Address	98:49:14:4F:B3:16
	Vehicle BT Name	BMW 92906
	Mobile Device Product Name	Galaxy S9+
	Mobile Device BT Name	SM-G965N
	Mobile Device BT Address	50:77:05:BE:37:CF
	Phone Book (Contact)	telcom/pb.vcf
Apple CarPlay	Vehicle BT Address	A0:56:B2:32:EB:23
	Vehicle BT Name	BMW 88366
	Vehicle Device ID	BC:30:7E:67:81:AB
	Vehicle Model Name	F25-NBTEvo-0716
	Mobile Device BT Name	syh2347′s iPhone
	Mobile Device Product Name	iPhone9, 3 (iPhone7)
	Mobile Device BT Firmware	BCM4355C0_17.1.199.5230_Elsa_OS _USI_MCC_TRS_20190827.hcd
	Mobile Device OS Version	13.3.1 (Build 17050)
	Mobile Device IMEI	355321083330481
	Mobile Device IMSI	450080020035886
	BT Pairing Pin	665367
	Link Layer Encryption Key	2822d5031202e3de820476958beaab8d
	Link Layer Encryption State	0x01

**Table 5:** List of Artifacts in Wireless Communication between the IVI and Mobile Device (Legacy System).

IVI System	Artifact	Details
Legacy System	Vehicle BT Address	A0:56:B2:32:EB:23
	Vehicle BT Name	BMW 88366
	Mobile Device Product Name	iPhone9, 3 (iPhone7)
	Mobile Device BT Firmware	BCM4355C0_17.1.199.5230_Elsa_OS _USI_MCC_TRS_20190827.hcd
	Mobile Device OS Version	13.3.1 (Build 17050)
	Mobile Device IMEI	355321083330481
	Mobile Device IMSI	450080020035886
	Link Layer Encryption Key	C93982dd761c1d63dcc467a9f90ac287
	Link Layer Encryption State	0x01
	Callee’s Phone Number	0107441xxxx
	Callee’s Name	20 2A AE 40 CC A0 C2 18 20 2C
	Caller’s Phone Number	0103680xxxx
	Caller’s Name	20 2A D6 4D AE 38 B3 D9 20 2C

**Table 6:** List of Artifacts in Mobile Device Internal Storage.

IVI System	Artifact	Details
Android Auto	Android Auto App List in Use	com.google.android.projection. gearhead.common.HOTSEAT.xml (Package Name)
	BT MAC Address of Paired Vehicle	common_user_settings.xml (MAC Address)
	Name of Paired Vehicle	common_user_settings.xml (Bluetooth Name)
	Last Used Time	app_state_shared_preferences.xml (UNIX Timestamp)
	Projected Activation Time	app_state_shared_preferences.xml (UNIX Timestamp)
	Disconnection Time	carservice.xml (UNIX Timestamp)
	Wi-Fi Connection History	Timestamp (MM-DD HH:MM:SS)
	Google Assistance History [13]	binarypb File
Apple CarPlay	BT MAC Address of Paired Vehicle	com.apple.carplay.plist (MAC Address)
	Name of Paired Vehicle	com.apple.carplay.plist (Bluetooth Name)
	Last Used Time	com.apple.carplay.plist (UNIX Timestamp)
	Last Siri Conversation	PreviousConversation.plist (Text)

**Table 7:** Internal storage specifications for BMW NBT HU EVO and Belsee Best Aftermarket Auto.

Specification	BMW NBT HU EVO	Belsee Best Aftermarket Auto
Type	Hard Disk Drive	NAND Flash
Capacity	200 GB	64 GB
Filesystem	QNX4, QNX6	Ext4, F2FS
OS	QNX	Android

**Table 8:** List of Artifacts in IVI System Internal Storage.

**IVI System**	**Artifact**	**Details**
Belsee Best Aftermarket Auto (Android Auto)	BT Paired Device List	bt_conf.ini (Bluetooth Name)
	Phone Number of BT Paired Device	DateBase1.db (Phone Number)
	Pairing Time of BT Paired Device	ivt.log (Unix Timestamp)
	BT Communication Log	SETTINGS.xml
	Vehicle Location	(Latitude, Longitude)

## Data Availability

Not applicable.
